# Modification of insulin amyloid aggregation by Zr phthalocyanines functionalized with dehydroacetic acid derivatives

**DOI:** 10.1371/journal.pone.0243904

**Published:** 2021-01-07

**Authors:** Svitlana Chernii, Yuriy Gerasymchuk, Mykhaylo Losytskyy, Damian Szymański, Iryna Tretyakova, Anna Łukowiak, Vasyl Pekhnyo, Sergiy Yarmoluk, Viktor Chernii, Vladyslava Kovalska

**Affiliations:** 1 Institute of Molecular Biology and Genetics, NASU, Kyiv, Ukraine; 2 Institute of Low Temperature and Structure Research, PAS, Wroclaw, Poland; 3 Institute of General and Inorganic Chemistry, NASU, Kyiv, Ukraine; Fondazione Pisana per la Scienza, ITALY

## Abstract

Amyloid fibrils are widely studied both as target in conformational disorders and as basis for the development of protein-based functional materials. The three Zr phthalocyanines bearing dehydroacetic acid residue (**PcZr(L1)**_**2**_) and its condensed derivatives (**PcZr(L2)**_**2**_ and **PcZr(L3)**_**2**_) as out-of-plane ligands were synthesized and their influence on insulin fibril formation was studied by amyloid-sensitive fluorescent dye based assay, scanning electron microscopy, fluorescent and absorption spectroscopies. The presence of Zr phthalocyanines was shown to modify the fibril formation. The morphology of fibrils formed in the presence of the Zr phthalocyanines differs from that of free insulin and depends on the structure of out-of-plane ligands. It is shown that free insulin mostly forms fibril clusters with the length of about 0.3–2.1 μm. The presence of Zr phthalocyanines leads to the formation of individual 0.4–2.8 μm-long fibrils with a reduced tendency to lateral aggregation and cluster formation (**PcZr(L1)**_**2**_), shorter 0.2–1.5 μm-long fibrils with the tendency to lateral aggregation without clusters (**PcZr(L2)**_**2**_), and fibril-like 0.2–1.0 μm-long structures (**PcZr(L3)**_**2**_). The strongest influence on fibrils morphology made by **PcZr(L3)**_**2**_ could be explained by the additional stacking of phenyl moiety of the ligand with aromatic amino acids in protein. The evidences of binding of studied Zr phthalocyanines to mature fibrils were shown by absorption spectroscopy (for **PcZr(L1)**_**2**_ and **PcZr(L2)**_**2**_) and fluorescent spectroscopy (for **PcZr(L3)**_**2**_). These complexes could be potentially used as external tools allowing the development of functional materials on protein fibrils basis.

## Introduction

The formation and deposition of ordered filamentous protein aggregates also known as amyloid fibrils are connected with a large number of human diseases, including amyloidoses and neurodegenerative disorders [1–3]. Amyloid fibrils are highly ordered cross-β sheet protein aggregates widely studied as target against conformational disorders, but due to the high stability they are also considered as basis for the development of functional materials. While the aspects of the inhibition of the pathological amyloid formation are studied in much detail up to date [4], the use of the functional self-assembling materials based on amyloid fibrils is still a growing field of research [5]. Amyloid fibrils are of high interest as functional materials due to a number of their unique properties such as high elasticity and stability, mechanical robustness, stiffness, easy assembly, the modulation of adhesion, etc. [6–8]. These functional materials could be applied as components in active composites, sensors and biomimetic structures or underwater adhesives [5, 6].

The mentioned reasons cause an interest in the search of compounds that could specifically detect amyloid fibrils and inhibit or modify fibril formation process. A number of molecules containing aromatic fragments are proposed as compounds that can bind to amyloid aggregates and, as a result, provide the fibril detection and/or influence the amyloid aggregation process.

Amyloid fibrils can be detected by fluorescence-based methods, using dyes sensitive to β-pleated grooves [9]. In this case, the commonly used dyes for the detection and visualization of amyloid aggregates are Thioflavin T [10] and Congo Red [11, 12]. Furthermore, compounds of cyanine [13] and styrylpyridinium [14] dye classes were proposed as probes for sensing of amyloid fibrils.

Recently, we have discovered β-ketoenoles as a new class of dyes possessing fluorescence sensitivity to amyloid fibrils [15, 16]. These compounds are able to specifically bind to the amyloid fibrils with the increase of fluorescent quantum yield and lifetime, while demonstrating a relatively weak response in the presence of native protein. β-ketoenole dye AmyGreen was also proposed as a stain for visualization of amyloids in bacterial biofilms of different amyloid-producing strains [17].

Dehydroacetic acid (DHA) is the basic material for the synthesis of a very wide range of organic compounds, in particular β-ketoenoles, described in many reviews [18–20]. DHA derivatives were studied for their antioxidant and cytotoxic [21], antitumor [22], antimicrobial [23–25] and other properties. The interaction of DHA with aromatic aldehydes leads to the formation of сhalcones [26, 27], which serve as starting compounds to obtain alkylamino-β-ketoenoles. Another feature of DHA and some of its derivatives is their ability to form coordination complexes with metals [28–30]. In the case of metals with high coordination numbers, the formation of mixed-ligand DHA complexes with 8-hydroxy quinoline [31] and β-diketones [32] is possible. The complexes of Zr and Hf phthalocyanines with these ligands have been also described [33–35].

Many external factors can strongly affect the stability of fibrils and the final structure of amyloid aggregates [36]. We have previously reported that the presence of macrocyclic metal complexes, namely out-of-plane coordinated phthalocyanines [37–39], planar phthalocyanines and porphyrazines [40] and tetraphenylporphyrins [41], are able to inhibit the fibril formation or significantly change the morphology of formed protein aggregates. Particularly, the presence of out-of-plane coordinated phthalocyanines led to the formation of different aggregate populations (small amount of protofilaments, oligomeric or unstructured amorphous aggregates) depending on the structure of their out-of-plane ligands [37–39]. Thus, the design of out-of-plane coordinated phthalocyanines as agents able to change fibril formation pathway seems to be an efficient way to develop a tool for design of functional materials based on amyloid fibrils.

**Fig 1:**
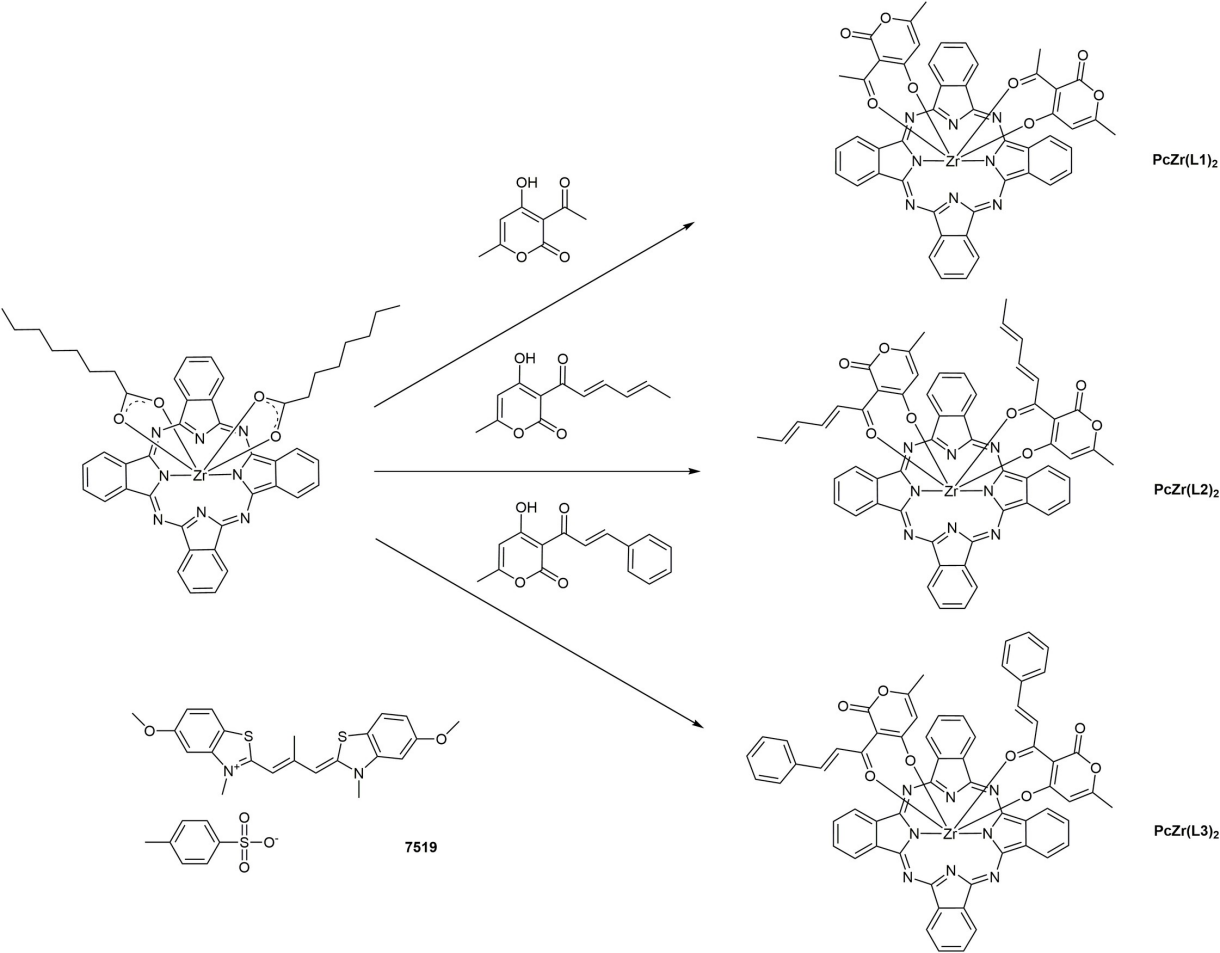
Structures of out-of-plane coordinated Zr phthalocyanines and amyloid-sensitive cyanine dye 7519.

Therefore, the aim of the work was to study Zr phthalocyanine complexes, which are structurally based on phthalocyanine core and bear DHA derivatives as out-of-plane ligands. According to this aim, three Zr phthalocyanines (ZrPc) bearing dehydroacetic acid (**PcZr(L1)**_**2**_) and its derivatives condensed with crotonaldehyde (**PcZr(L2)**_**2**_) and benzaldehyde (**PcZr(L3)**_**2**_) as out-of-plane coordinated ligands (Fig 1) were synthesized. These ligands are related to the amyloid-binding β-ketoenole dyes, for which amyloid-sensitive properties have previously been shown [15, 16]. We thus suppose that DHA derivatives as out-of-plane ligands could play role in the binding of phthalocyanines to amyloid fibrils. Thus, the effect of Zr phthalocyanines on the kinetics of amyloid insulin aggregation was studied and the morphology of insulin aggregates formed in presence of Zr phthalocyanines was determined by scanning electron microscopy (SEM). Finally, we used UV-VIS absorption and fluorescence methods to study the binding of phthalocyanines with native insulin and its mature amyloid fibrils.

## Material and methods

### Materials

Human insulin was acquired from Sigma-Aldrich. Prof. O. I. Tolmachev and Dr. Yu. L. Slominskii provided amyloid-sensitive cyanine dye 7519 (Institute of Organic Chemistry of NASU). Dimethyl sulfoxide (DMSO), methanol (MeOH), 0.1 M HCl solution in water, and 50 mM Tris-HCl buffer (pH 7.9) were used as solvents.

### The general method of synthesis of L2 and L3 ligands (Scheme 1)

To 10 mmol of dehydroacetic acid in 10 mL of butanol, equimolar amount of aldehyde was added and the solution was heated to 80°C. 3 drops of a pyridine mixture with piperidine (1:1 by volume) were added to the boiling homogeneous solution and boiled for 3 hours. When half of the solvent was distilled, the mixture was cooled and derived crystals were filtered. The product was washed twice on the filter with small amount of isopropanol and recrystallized from dimethylformamide-isopropanol system. Then, it was filtered, washed on the filter with isopropanol, then washed twice with water and finally air-dried.

**Scheme 1:**
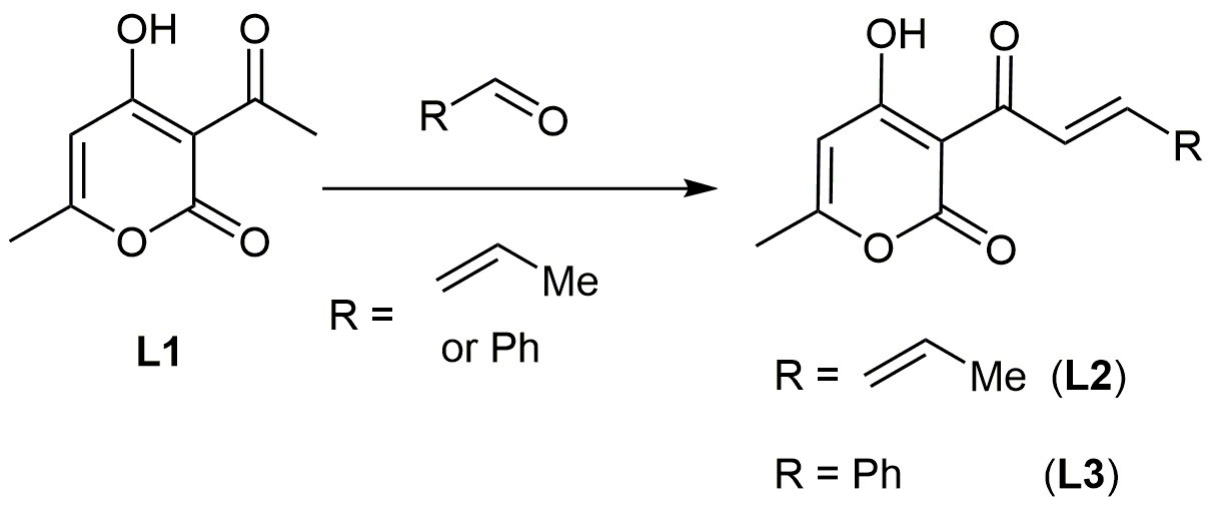
Synthesis scheme of condensed derivatives of dehydroacetic acid.

**L2.** 3-(2E,4E)-hexa-2,4-dienoyl-4-hydroxy-6-methyl-2H-pyran-2-one. Yield: 45%. M.p. = 150–154°C. Found (%): C, 65.53; H, 5.41. Anal. Calcd. (%) for C_12_H_12_O_4_: C, 65.45; H, 5.49. ^1^H NMR (400 MHz, CDCl_3_) δ 18.08 (s, 1H), 7.59 (p, *J* = 15.0 Hz, 2H), 6.62–6.15 (m, 2H), 5.92 (s, 1H), 2.26 (s, 3H), 1.92 (d, *J* = 6.0 Hz, 3H).

**L3.** 3-cinnamoyl-4-hydroxy-6-methyl-2H-pyran-2-one. Yield: 78%. M.p. = 136–137 ^o^C. Found (%): C, 70.39; H, 4.75. Anal. Calcd. (%) for C_15_H_12_O_4_: C, 70.31; H, 4.72. ^1^H NMR (400 MHz, CDCl_3_) δ 17.95 (s, 1H), 8.32 (d, *J* = 15.7 Hz, 1H), 7.97 (d, *J* = 15.7 Hz, 1H), 7.74–7.64 (m, 2H), 7.48–7.30 (m, 3H), 5.97 (d, *J* = 0.9 Hz, 1H), 2.29 (d, *J* = 0.9 Hz, 3H).

### The general method of synthesis of phthalocyanine complexes of zirconium with dehydroacetic acid (L1) and its derivatives (L2, L3) as out-of-plane coordinated ligands

445 mg of РсZr(C_7_H_15_COO)_2_ (obtained as described in [42]) (0.5 mmol) was dissolved in 3 mL of toluene under heating; 1.2 mmol of dehydroacetic acid or its derivatives (20% excess) was also dissolved in hot toluene (2 mL). Reagents were mixed and refluxed for 4 hours, and then reaction mixture was cooled. The resulting crystalline precipitate was filtered and washed with large amount of acetone. Product was dried at 60°C.

#### PcZr(L1)_2_

Bis-[3-acetyl-4-hydroxy-6-methyl-2H-pyran-2-onato] zirconium phthalocyaninate. Yield: 75%. Found (%): Zr 9.62. Anal. Calcd. (%) for C_48_H_30_N_8_O_8_Zr: Zr, 9.72. ^1^H NMR (400 MHz, CDCl_3_) δ 9.46–9.33 (m, 4H), 9.27 (d, *J* = 7.5 Hz, 2H), 9.10 (t, *J* = 7.0 Hz, 2H), 8.55–7.82 (m, 8H), 4.71 (d, *J* = 8.9 Hz, 2H), 1.97 (s, 6H), 1.76–1.64 (m, 6H).

#### PcZr(L2)_2_

Bis-[3-(2E,4E)-hexa-2,4-dienoyl-4-hydroxy-6-methyl-2H-pyran-2-onato] zirconium phthalocyaninate. Yield: 27%. Found (%): Zr, 8.91. Anal. Calcd. (%) for C_56_H_38_N_8_O_8_Zr: Zr, 8.75. ^1^H NMR (400 MHz, CDCl_3_) δ 9.57–9.00 (m, 8H), 8.32–7.93 (m, 8H), 6.60 (t, *J* = 17.7 Hz, 2H), 6.20–5.93 (m, 2H), 5.87 (t, *J* = 12.3 Hz, 2H), 5.44–5.17 (m, 3H), 4.79 (s, 1H), 1.99 (s, 6H), 1.76 (d, *J* = 6.7 Hz, 6H).

#### PcZr(L3)_2_

Bis-[3-cinnamoyl-4-hydroxy-6-methyl-2H-pyran-2-onato] zirconium phthalocyaninate. Yield: 72%. Found (%): Zr, 8.24. Anal. Calcd. (%) for C_62_H_38_N_8_O_8_Zr: Zr, 8.19. ^1^H NMR (400 MHz, CDCl_3_) δ 9.90–8.79 (m, 8H), 8.64–7.72 (m, 8H), 7.72–7.09 (m, 10H), 6.97–6.86 (m, 2H), 6.61–6.01 (m, 2H), 5.05–4.46 (m, 2H), 2.16–1.32 (m, 6H).

### Preparation of stock solutions

The insulin solution with concentration of 2 mg/mL (340 μM) was prepared by dissolving the weighted amount of human insulin in 0.1 M HCl solution in distilled water. The stock solutions of phthalocyanines (2 mM) were prepared in DMSO.

### Insulin fibril formation

Non-inhibited insulin fibrils were formed by incubating the 340 μM protein solution in a water bath at 65°C for about 5 h. To obtain fibrils inhibited by the studied phthalocyanines, an aliquot of 2 mM DMSO solution of corresponding phthalocyanine was added to the 340 μM protein solution (final concentration of phthalocyanine in reaction mixture was 100 μM); the obtained mixture was than incubated in water bath at 65°C for about 5 h. The experiment was repeated 4 times.

### Monitoring of amyloid fibril formation

The kinetics of the fibrillization reaction was monitored using the fluorescent dye 7519 that provides specific fluorescence response in the presence of amyloid fibrils. This assay was previously developed by us [43] and applied in similar studies [37–40]; we suppose the fluorescence intensity of the dye to be the measure of the quantity of beta‐pleated amyloid structures. For this, aliquots of the reaction mixture were withdrawn from each tube at about 90, 150, 240, and 300 minutes after the reaction started, and added to 2 μM solution of 7519 in 50 mM Tris-HCl buffer (pH 7.9). Fluorescence emission spectrum of 7519 (excitation at 580 nm) was measured immediately after mixing the protein and dye solutions with the help of fluorescent spectrophotometer Cary Eclipse (Varian, Australia). The efficiency of inhibition of fibrillization reaction by each phthalocyanine was estimated as (1–I/I_0_)×100%, where I and I_0_ are the fluorescence intensities of 7519 measured in the presence of inhibited and non-inhibited fibrils after 300 minutes of incubation. Average values of inhibition efficiency (calculated based on 4 repeats of the experiment) are provided, along with standard deviation represented as error bars.

### Scanning electron microscopy study

SEM studies of the products of fibrillization reaction of insulin in the absence and in the presence of the studied compounds were carried out using FE-SEM microscope (FEI Nova NanoSEM 230) equipped with an EDS analyzer (EDAX Genesis XM4). SEM images were recorded using an accelerating voltage of 5.0 kV. For layer deposition, the samples of insulin amyloid aggregates obtained at the concentration of 340 μM were diluted in 15 times with distilled water. Then, a drop of the solution was deposited on the silicon surface. The samples were studied after water evaporation. The length of the fibrils was determined using Gwyddion program.

### UV-VIS spectroscopy study

Absorption spectra of phthalocyanines PcZr(L1)_2_, PcZr(L2)_2_, PcZr(L3)_2_ and ligands L1, L2, L3 with insulin were recorded on a SHIMADZU UV-VIS-NIR spectrophotometer UV-3600. Spectra were recorded at 250–820 nm region. Monomeric insulin and noninhibited insulin fibrils (after 300 min of reaction) at concentration of 340 μM were diluted in 20 times in 50mM Tris-HCl buffer (pH 7.9) and then the aliquot of stock solution (2 mM in DMSO) of corresponding compound (phthalocyanine or ligand) was added, so that the compound’s final concentration was 5 μM (thus the compound-to-protein concentrations ratio was the same as during the fibril formation reaction). Working solutions of phthalocyanines and ligands in methanol were also 5 μM. All spectral measurements were performed in quartz absorption cuvettes (1 ґ 1 cm) at room temperature.

### Fluorescent spectroscopy study

The evaluation of fluorescent sensitivity of phthalocyanines and ligands to monomeric insulin and mature fibrils was also performed. Fluorescence excitation and emission spectra were collected on a Cary Eclipse fluorescence spectrophotometer (Varian, Austria). Working solutions of the compounds (phthalocyanines and ligands) were prepared by dilution of the compound’s stock solution with Tris-HCl buffer (pH 7.9) to the concentration of 2 μM (for all measurements in buffer) with the further μligands in methanol were 5 μM. All spectral measurements were performed in standard quartz cuvettes (1 ґ 1 cm) at room temperature.

## Results and discussion

### The synthesis of zirconium phthalocyanines

Dehydroacetic acid [44] and its derivatives [45] are chelating bidentate ligands exhibiting properties corresponding to those of β-diketones and quite easily forming complexes with boron [46] and transition metals [47]. According to the Knoevenagel reaction, we obtained dehydroacetic acid derivatives condensed with aldehydes, which (similarly to DHA) have ketoenole fragments suitable for coordination [47]. These derivatives also interact with coordination-unsaturated central metal atom of phthalocyanines (Fig 1) and form out-of-plane complexes. In this work, we have obtained phthalocyanine complexes with dehydroacetic acid (**PcZr(L1)**_**2**_) and its condensed derivatives (**PcZr(L2)**_**2**_ and **PcZr(L3)**_**2**_). The resulting complexes are highly stable fine crystalline substances of dark blue color, with absorption bands around 330 nm (Soret band) and 690 nm (Q-band). The yields for these reactions are about 30–75%. The studied complexes are soluble in most organic solvents.

### Fluorescent dye-based assay study of fibril formation kinetics

**Fig 2:**
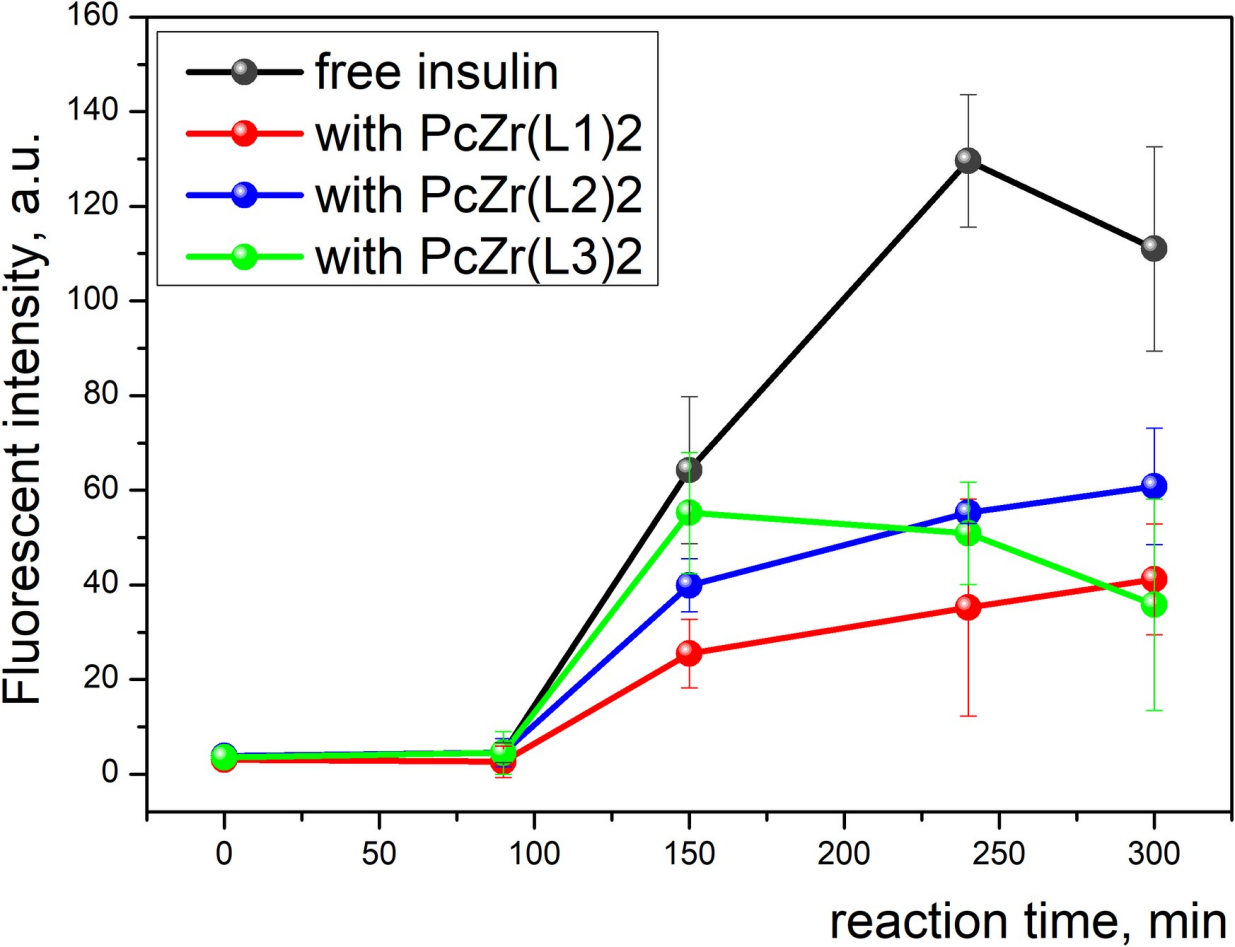
The kinetics of insulin fibrillization reaction under the influence of Zr phthalocyanines bearing different ligands.

Insulin is widely used as a model protein in the study of amyloid aggregation. Moreover, amyloid deposits of fibrillar insulin have been reported in patients with diabetes [48], during normal aging [49], and after repeated injections of insulin [50]. The monitoring of kinetics of the insulin fibril formation (Fig 2) under the influence of out-of-plane coordinated Zr phthalocyanines was studied by fluorescent assay based on amyloid-sensitive cyanine dye 7519. The presence of phthalocyanines slightly affects the fluorescence intensity of the cyanine dye 7519 (S1 Fig). The dye emission intensity starts to increase in 90 min after the beginning of reaction for free insulin as well as in the presence of all three compounds. Thus, the presence of phthalocyanines does not change the duration of the lag phase of the kinetics, which may indicate that the studied phthalocyanines do not significantly affect the early stages of aggregation, the so-called nucleation (the formation of stable association nuclei) [51]. The shapes of the kinetics curves are similar for compounds **PcZr(L1)**_**2**_ and **PcZr(L2)**_**2**_, while it differs for **PcZr(L3)**_**2**_. We suggest that aggregation intermediates intensively formed on the early stages of insulin aggregation in the presence of **PcZr(L3)**_**2**_ partially degrade during the later elongation stages. The efficiency of inhibition at the final stage of the reaction intensity was found to be in the range from 45% for compound **PcZr(L2)**_**2**_ to 68% for compound bearing two chalcone ligands (**PcZr(L3)**_**2**_). Thus, dye-based fluorescent assay shows the difference in the effect of these phthalocyanines on the kinetics of insulin aggregation, which may indicate the difference in an amount of formed insulin aggregates and/or their morphology.

### Scanning electron microscopy study of the morphology of insulin aggregates

**Fig 3:**
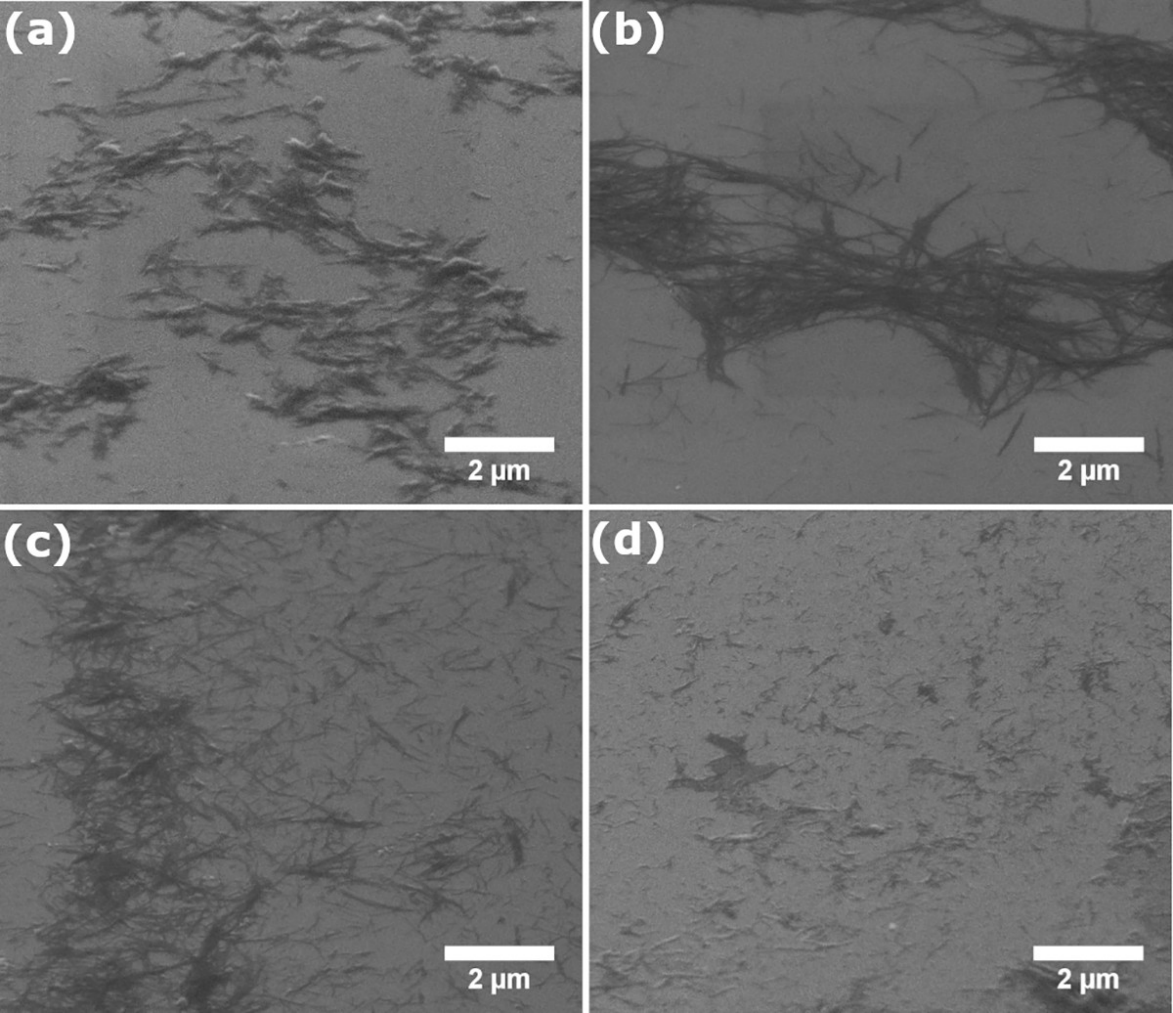
SEM images of insulin fibrils formed in the absence of phthalocyanines (a) and in the presence of PcZr(L1)_2_ (b), PcZr(L2)_2_ (c), and PcZr(L3)_2_ (d).

The morphology of aggregates formed by insulin in free state and under the influence of out-of-plane coordinated Zr phthalocyanines was determined by method of scanning electron microscopy (Fig 3, Table 1). Different chemical nature of the out-of-plane ligands may affect phthalocyanine binding ability and provide distinctions in morphology of insulin amyloid aggregates. Free insulin forms separate fibrils with length of about 0.2–2.1 μm sticking in large clots (bundles) and clusters (Fig 3A). The wide spread of laterally aggregated fibril clusters indicates a high tendency of free insulin fibrils to form such structures. Only a small number of single fibrils are present in the images (Fig 3A). At the same time, the presence of the studied Zr phthalocyanines induces changes in aggregate structures leading to the formation of fibrils with substantially different morphology.

**Table 1:** Parameters of insulin amyloid fibrils formed in the presence of Zr phthalocyanines.

Name of ZrPc	Morphology of products	Length (range), μm	Length (average*), μm
Free insulin	fibril cluster	0.3–2.1	0.9±0.4
**PcZr(L1)**_**2**_	mature fibril	0.4–2.8	1.6±0.25
**PcZr(L2)**_**2**_	mature fibril	0.2–1.5	0.6±0.35
**PcZr(L3)**_**2**_	fibril-like structure	0.2–1.0	0.4±0.2

*Calculated based on 20 measurements of fibrils length.

Thus, the presence of **PcZr(L1)**_**2**_ leads to the formation of individual 0.4–2.8 μm-long mature fibrils with a reduced tendency to lateral aggregation and cluster formation (Fig 3B). These fibrils are longer than in the case of free insulin. In contrast to the free insulin and **PcZr(L1)**_**2**_ presence, **PcZr(L2)**_**2**_ leads to the formation of shorter fibrils with a tendency to lateral aggregation, but without the formation of clusters (Fig 3C). It should be mentioned that this Zr phthalocyanine has the lowest effect on the kinetics of insulin aggregation according to the fluorescence assay. Comparison of the effects of **PcZr(L1)**_**2**_ and **PcZr(L2)**_**2**_ presence shows that in the latter case separate fibrils dominated over lateral assembles of fibrils. Finally, in the case of **PcZr(L3)**_**2**_ we observed the formation of fibril-like structures and «clots» 0.2–1.0 nm in size for which it is difficult to determine their specific morphology (Fig 3D).

It was previously shown that phthalocyanine dichloride with small-size substituents redirects the amyloid aggregation process towards the formation of large size particles (diameter up to 100 nm and higher) that were attributed to amorphous aggregates [38]. At the same time, the presence of the studied phthalocyanines containing DHA and its derivatives as the out-of-plane ligands affects the type of aggregation product so that the formation of fibrils is observed. This allows us to suppose that chemical nature of out-of-plane ligands of phthalocyanines is to the large extend responsible for the changes in insulin fibrils morphology. The phthalocyanines bearing DHA (methyl side group) or DHA derivatives with alken side group (**PcZr(L1)**_**2**_ and **PcZr(L2)**_**2**_, correspondingly) redirect aggregation reaction to the formation of long separate filaments. At the same time, the compound **PcZr(L3)**_**2**_ bearing aromatic residue lead to the formation of short aggregates with a tendency to gluing into clots.

### VIS absorption study of Zr phthalocyanines and their ligands

**Fig 4:**
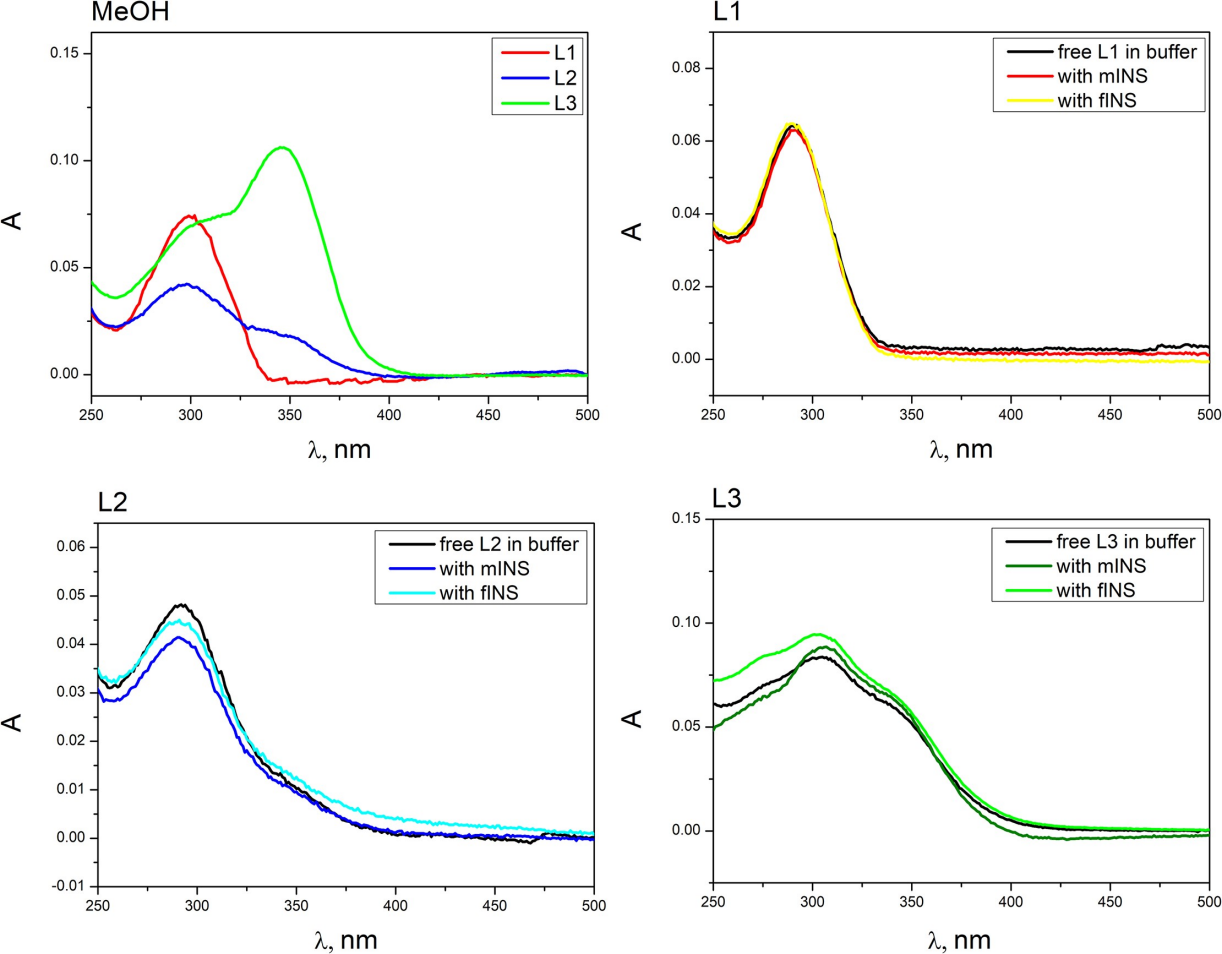
Absorption spectra of ligands in MeOH (a) and in Tris-HCl buffer pH 7.9 and in the presence of monomeric (mINS) and fibrillar(fINS) insulin (L1 (b)_,_ L2 (c), and L3 (d).

**Fig 5:**
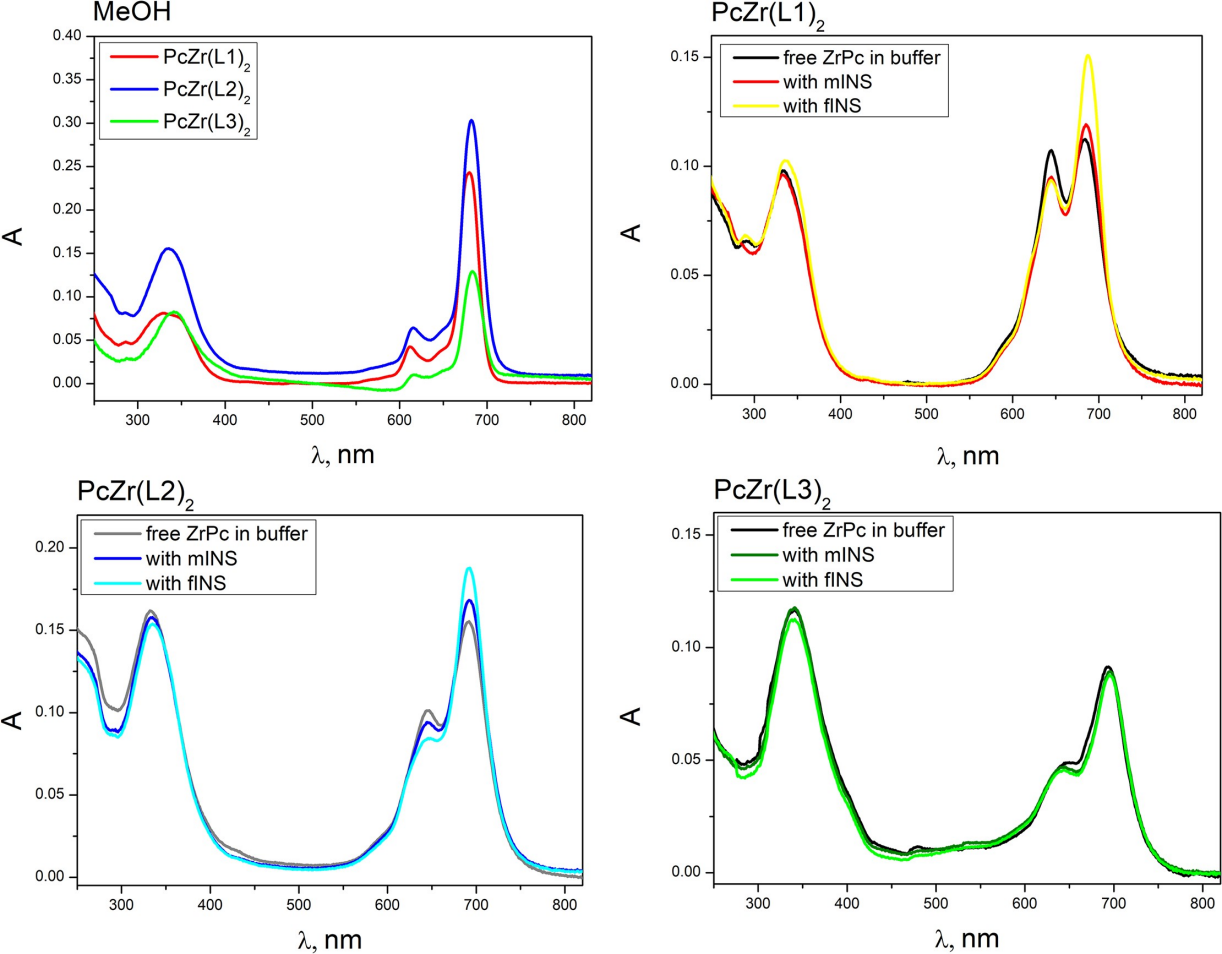
Absorption spectra of phthalocyanines in MeOH (a) and in Tris-HCl buffer pH 7.9, in the presence of monomeric (mINS) and fibrillar (fINS) insulin (PcZr(L1)_2_ (b)_,_ PcZr(L2)_2_ (c), and PcZr(L3)_2_ (d).

**Fig 6:**
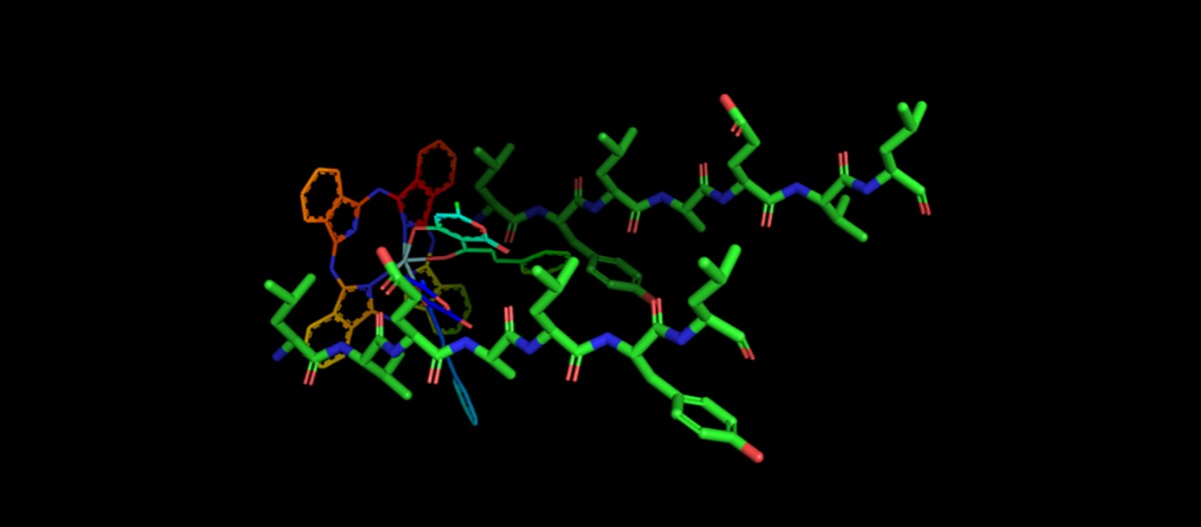
Illustrative scheme of PcZr(L3)_2_ binding to antiparallel β-sheet groove of insulin amiloidogenic sequences (11)LVEALYL(17).

In order to study the binding of phthalocyanines with native protein and mature fibrils, the spectral-luminescent methods were used. Thus, the study of UV-VIS absorption behavior of ligands L1, L2, L3 and corresponding Zr phthalocyanines PcZr(L1)_2,_ PcZr(L2)_2_, and PcZr(L3)_2_ in MeOH and Tris-HCl buffer (pH 7.9) was performed (Figs 5 and 6). For L1 in MeOH, the band with a maximum at 300 nm is observed, L2 has the broad band with the maximum at the same wavelength as L1 (Fig 4A). Meanwhile, the maximum of the L3 absorption spectrum in MeOH is located at 346 nm. Absorption spectra of ligands in buffer contain a band with maximum at 291–292 nm for L1 and L2, and 305 nm for L3. The UV-VIS absorption behavior of ligands (L1, L2, L3) in the presence of monomeric and fibrillar protein was studied in order to detect their interaction (Fig 4B–4D). By this method, no interaction between ligands and protein was observed.

Fluorescence spectroscopy was also used to study the properties of ligands (S1 Table). In MeOH, compounds L1 and L2 have a maximum of fluorescence emission at 340 nm and a maximum of fluorescence excitation at 265 nm. L3 is characterized by fluorescence emission and excitation maxima at 491 nm and 391 nm respectively. These compounds do not possess fluorescence in buffer as well as in the presence of proteins.

It was previously shown that phthalocyanine complexes with pronounced tendency to self-association have a higher anti-fibrillogenic activity [4, 39, 52]. The interaction between aromatic amino acids of protein and macrocycle core of phthalocyanine *via* π-stacking provides a central mechanistic basis for the inhibitory activity of these compounds [53]. Since self-aggregation of phthalocyanine is also provided by π-stacking interactions [54], the higher tendency of the compound to self-aggregation provides better binding of phthalocyanine to aromatic amino acids of the protein [4].

Previously, the destruction of self-associates of phthalocyanine dichloride (containing small-size chlorine atoms as substituents) in the presence of insulin fibrils has been shown, which may indicate an interaction between the compound and the protein [39]. Moreover, the enhancement of the intensity in phthalocyanine dichloride surface-enhanced Raman scattering spectra caused by the presence of insulin fibrils was observed that may reflect the interaction between phthalocyanine complex and amyloid fibrils [55].

In order to estimate the self-association behavior of the studied phthalocyanines **PcZr(L1)**_**2**_, **PcZr(L2)**_**2**_ and **PcZr(L3)**_**2**_, we have studied absorption spectra of these compounds in MeOH and buffer (Fig 5). Thus in the compounds’ spectra in MeOH, intensive long-wavelength peak of Q-band with maximum at 680–684 nm was observed along with a short-wavelength satellite band with a maximum at 612–616 nm (Fig 5A). Another characteristic feature of phthalocyanines’ electronic absorption spectra is the Soret band, which is situated at 334–342 nm for the studied compounds. Besides, in the spectrum of phthalocyanines **PcZr(L1)**_**2**_ and **PcZr(L2)**_**2**_ in MeOH there is a ligand band at 285 nm. Meanwhile, the ligand band of **PcZr(L3)**_**2**_ appears to overlap with the Soret band of phthalocyanine, since the ligand maximum is located at 373 nm (while the maximum of the Soret band is at 342 nm). According to the literature, phthalocyanines exist as monomeric species in methanol at 5 μM concentration revealing the observed shape of the absorption spectrum [54]. Thus, ZrPc spectra in MeOH correspond to the spectra of unassociated phthalocyanines (in their monomeric form).

Absorption spectra of Zr phthalocyanines in buffer contain two Q-band peaks, namely short-wavelength band Q_2_ near 645 nm (at least partially corresponding to aggregates) and long-wavelength one Q_1_ in the range 684–695 nm, as well as Soret band at 333–339 nm (Fig 5B–5D). The ratio between the intensities of short- and long-wavelength Q-bands point to the conclusion that free **PcZr(L1)**_**2**_ (Fig 5B) has a higher tendency for aggregation compared to **PcZr(L2)**_**2**_ and **PcZr(L3)**_**2**_. The presence of both monomeric and fibrillar insulin does not change the shape of **PcZr(L3)**_**2**_ spectra (Fig 5D). At the same time, an addition of fibrillar insulin to the **PcZr(L1)**_**2**_ solution leads to the significant decrease of short-wavelength Q-band and increase of long-wavelength one, which indicates the destruction of phthalocyanines aggregates (self-associates) accompanied with the release of monomer phthalocyanines (Fig 5B). Although **PcZr(L2)**_**2**_ has lower tendency to self-association (compared to **PcZr(L1)**_**2**_), for this phthalocyanine the similar redistribution of the Q-bands intensity upon addition of insulin fibrils was shown. We can thus assume that phthalocyanines **PcZr(L1)**_**2**_ and **PcZr(L2)**_**2**_ interact with mature fibrils of insulin. The less intensive destruction of self-associates of **PcZr(L1)**_**2**_ and **PcZr(L2)**_**2**_ is also observed in the presence of monomeric insulin, which indicates that the compounds have a higher tendency to interaction with fibrillar insulin as compared to monomer one.

The spectral-luminescent properties of Zr phthalocyanines have also been studied by fluorescence spectroscopy. Excitation maxima of phthalocyanines are located at 688–693 nm with emission in the range 697–700 nm. No increase or decrease in the fluorescence intensity of Zr phthalocyanines in the presence of the initial and final products of amyloid aggregation was observed for **PcZr(L1)**_**2**_ and **PcZr(L2)**_**2**_, while increase in 2.0 times for **PcZr(L3)**_**2**_ was registered upon addition of fibrillary insulin (S1 Table). This observation is considered to reflect the binding of **PcZr(L3)**_**2**_ with mature fibrils. Thus, the spectral methods point to the interaction of compounds **PcZr(L1)**_**2**_, **PcZr(L2)**_**2**_, and **PcZr(L3)**_**2**_ with mature amyloid fibrils.

### Discussion of mechanism of activity

Studied Zr phthalocyanines with out-of-plane coordinated ligands are considered as compounds able to modify the fibril formation reaction. We observed that the presence of phthalocyanines led to the formation of fibrils of different structure depending on the structure of out-of-plane ligands. The formation of protein aggregates of other types such as amorphous or oligomeric species was not observed (Fig 3). We presume it is happening due to the insignificant effect of the Zr phthalocyanines at the early stages (seed formation stage) of fibril formation reaction. However, morphology of formed filaments is changed by the phthalocyanines at later stages (probably elongation stages).

It is known that the structural basis for the influence of phthalocyanines on amyloid fibrils aggregation relies on specific π−π interactions between the aromatic ring system of these molecules and aromatic residues in the amyloidogenic proteins [4, 56, 57]. Insulin molecule contains tyrosine aromatic residues (Y) in amyloidogenic regions of protein in both chains—(13)L**Y**QLEN(18) in A-chain and (11)LVEAL**Y**L(17) in B-chain [58, 59]. Since the studied phthalocyanines differ only by their out-of-plane ligands, the main difference in the effect of Zr phthalocyanines on insulin aggregation should be contributed to these ligands. First, it can be assumed that Zr phthalocyanines may be able to bind into β-sheet grooves of growing fibrils with their out-of-plane ligands, since these ligands are a “core” of amyloid-specific dyes that bind to these sites. The strongest influence on fibrils morphology by **PcZr(L3)**_**2**_ could be explained by the presence of phenyl moiety in the out-of-plane ligand. It also explains the lower effect of **PcZr(L2)**_**2**_ since it contains a ligand of the similar structure as **PcZr(L3)**_**2**_ but without aromatic fragment. Thus, the presence of aromatic fragment in ligand may provide an additional fixation (*via* stacking to aromatic aminoacid residue) during the compound-protein interaction. That, in turn, may block protein-protein interaction and further fibril growth. It can be assumed that phthalocyanine interacts with a growing fibril in β-folds, forming an aromatic bond between the phthalocyanine core or phenyl fragment of coordinated ligand and tyrosine amino acid residue (Y14 in A-chain or Y17 in B-chain) (Fig 6). It should be noted that in the case of antiparallel folding of β-sheets in amyloid fibril of insulin, the possible option is binding of phthalocyanine simultaneously with two tyrosine residues in antiparallel chains (*via* stacking interactions of both macrocycle core and coordinated ligand).

## Conclusions

Three Zr phthalocyanines bearing dehydroacetic acid (**PcZr(L1)**_**2**_) and its derivatives (**PcZr(L2)**_**2**_**, PcZr(L3)**_**2**_) as out-of-plane ligands was synthesized and studied for their influence on insulin fibril formation. Monitoring of the kinetics of insulin fibrillization showed that the presence of phthalocyanines decreases the intensity of this reaction, which varies depending on the structure of out-of-plane ligand. This decrease was estimated by fluorescent assay as 45, 63 and 68% of initial intensity for compounds **PcZr(L2)**_2_, **PcZr(L1)**_**2**_), and **PcZr(L3)**_**2**_, respectively.

The morphology of fibrils formed in the presence of the Zr phthalocyanines differs from that of free insulin and also depends on the structure of out-of-plane ligands. Free insulin mostly forms fibril clusters with length of about 0.3–2.1 μm. The presence of Zr phthalocyanines leads to the formation of individual 0.4–2.8 μm-long fibrils with a reduced tendency to lateral aggregation and cluster formation for **PcZr(L1)**_**2**_, shorter 0.2–1.5 μm-long fibrils with a tendency to lateral aggregation without clusters for **PcZr(L2)**_**2**_, and fibril-like structures of 0.2–1.0 μm in length for **PcZr(L3)**_**2**_. The strongest influence on fibrils morphology by **PcZr(L3)**_**2**_ could occur due to the stacking of phenyl moiety of the out-of-plane ligand with side chain aromatic residues of amino acids.

The spectral methods show the evidence of the interaction between **PcZr(L1)**_**2**_, **PcZr(L2)**_**2**_, and **PcZr(L3)**_**2**_ and mature amyloid fibrils. Thus, the presence of mature fibrils leads to an increase in the Q-band absorption corresponding to phthalocyanine monomers (Q_1_) and a decrease in the intensity of Q-band of aggregates (Q_2_), which indicates the destruction of self-associates of **PcZr(L1)**_**2**_ and **PcZr(L2)**_**2**_. The presence of amyloid fibrils leads to an increase of the **PcZr(L3)**_**2**_ fluorescence intensity (in 2 times) that probably reflects its binding to amyloid fibrils.

Reported Zr phthalocyanines are suggested as agents with well-pronounced modifying activity for insulin aggregation reaction; they cause a decrease in reaction intensity and changes in the morphology of formed aggregates. Thus they are considered to be of interest as a potential tool for the design of functional materials based on protein amyloid fibrils.

## Supporting information

S1 Fig The fluorescence spectra of 7519 with PcZr(L1)2, PcZr(L2)2, PcZr(L3)2 in the absence and in the presence of insulin fibrils. (DOCX)

S1 Table Spectral-luminescent properties of studied ZrPc in free state and in presence of monomeric insulin and mature fibrils. (DOCX)

S2 Table Spectral-luminescent properties of studied compounds in MeOH. (DOCX)
