# Supplementary material for: Modification of insulin amyloid aggregation by Zr phthalocyanines functionalized with dehydroacetic acid derivatives
Source: PLoS One. 2021 Jan 7;16(1):e0243904. doi: 10.1371/journal.pone.0243904 (PMC7790233; doi:10.1371/journal.pone.0243904)
Supplement: S1 Fig — (DOCX) [file pone.0243904.s001.docx]

Figure S1. The fluorescence spectra of 7519 with PcZr(L1)_2_, PcZr(L2)_2_, PcZr(L3)_2_ in the absence and in the presence of insulin fibrils.
