# Supplementary material for: Modification of insulin amyloid aggregation by Zr phthalocyanines functionalized with dehydroacetic acid derivatives
Source: PLoS One. 2021 Jan 7;16(1):e0243904. doi: 10.1371/journal.pone.0243904 (PMC7790233; doi:10.1371/journal.pone.0243904)
Supplement: S1 Table — (DOCX) [file pone.0243904.s002.docx]

**Table S1 Spectral-luminescent properties of studied ZrPc in free state and in presence of monomeric insulin and mature fibrils.**

| Compound in Tris-HCl buffer | Free state | | | With mINS | | | With fINS | | |
| --- | --- | --- | --- | --- | --- | --- | --- | --- | --- |
|  | λ_ex_, nm | λ_em_, nm | I, a.u. | λ_ex_, nm | λ_em_, nm | I, a.u. | λ_ex_, nm | λ_em_, nm | I, a.u. |
| PcZr(L1)_2_ | 688 | 697 | 148 | 688 | 697 | 161 | 688 | 697 | 139 |
| PcZr(L2)_2_ | 688 | 698 | 280 | 688 | 698 | 227 | 688 | 698 | 311 |
| PcZr(L3)_2_ | 693 | 700 | 40 | 693 | 699 | 55 | 691 | 698 | 78 |
