# Supplementary material for: Modification of insulin amyloid aggregation by Zr phthalocyanines functionalized with dehydroacetic acid derivatives
Source: PLoS One. 2021 Jan 7;16(1):e0243904. doi: 10.1371/journal.pone.0243904 (PMC7790233; doi:10.1371/journal.pone.0243904)
Supplement: S2 Table — (DOCX) [file pone.0243904.s003.docx]

**Table S2 Spectral-luminescent properties of studied compounds in MeOH**

| Compound in MeOH | Free state | | | Compound  in MeOH | Free state | | |
| --- | --- | --- | --- | --- | --- | --- | --- |
| L1 | 265 | 340 | 39.9 | PcZr(L1)_2_ | 682 | 692 | 358 |
| L2 | 265 | 340 | 35.5 | PcZr(L2)_2_ | 684 | 691 | 757 |
| L3 | 331 | 430 | 55.4 | PcZr(L3)_2_ | 684 | 691 | 119.7 |

***λex. (λem.)*** *– maximum wavelength of fluorescence excitation (emission) spectrum,* ***I*** *- emission intensity of compounds,* ***a.u.*** *– arbitrary units, fINS and mINS – fibrillar and monomeric insulin, respectively.*
